# Upregulation of Heme Oxygenase-1 in Response to Wild Thyme Treatment Protects against Hypertension and Oxidative Stress

**DOI:** 10.1155/2016/1458793

**Published:** 2016-09-28

**Authors:** Nevena Mihailovic-Stanojevic, Zoran Miloradović, Milan Ivanov, Branko Bugarski, Đurđica Jovović, Danijela Karanović, Una-Jovana Vajić, Draženka Komes, Jelica Grujić-Milanović

**Affiliations:** ^1^Institute for Medical Research, University of Belgrade, Dr. Subotica 4, P.O. Box 102, 11129 Belgrade, Serbia; ^2^Faculty of Technology and Metallurgy, Department of Chemical Engineering, University of Belgrade, Karnegijeva 4, Belgrade, Serbia; ^3^Faculty of Food Technology and Biotechnology, University of Zagreb, Pierottijeva 6, 10 000 Zagreb, Croatia

## Abstract

High blood pressure is the most powerful contributor to the cardiovascular morbidity and mortality, and inverse correlation between consumption of polyphenol-rich foods or beverages and incidence of cardiovascular diseases gains more importance. Reactive oxygen species plays an important role in the development of hypertension. We found that wild thyme (a spice plant, rich in polyphenolic compounds) induced a significant decrease of blood pressure and vascular resistance in hypertensive rats. The inverse correlation between vascular resistance and plasma heme oxygenase-1 suggests that endogenous vasodilator carbon monoxide generated by heme oxidation could account for this normalization of blood pressure. Next product of heme oxidation, bilirubin (a chain-breaking antioxidant that acts as a lipid peroxyl radical scavenger), becomes significantly increased after wild thyme treatment and induces the reduction of plasma lipid peroxidation in hypertensive, but not in normotensive rats. The obtained results promote wild thyme as useful supplement for cardiovascular interventions.

## 1. Introduction

High blood pressure is certainly the most prevalent and powerful contributor to the cardiovascular morbidity and mortality in the majority of industrialized countries, with essential hypertension accounting for about 95% of all cases of hypertension [1]. Hypertension is recognized as a highly significant risk factor, and many effective antihypertensive drugs are developed, including angiotensin converting enzyme inhibitors, angiotensin-II receptor antagonists, diuretics, beta blockers, calcium channels blockers, and nitric oxide (NO) donors [2, 3].

Yet, for various reasons, hypertension is still a poorly controlled disorder, even in countries with very efficient preventive medical services. At the same time, about 75 to 80% of the world population (particularly in developing countries) uses alternative treatment options, often herbal medicines for the treatment of various health problems [4]. It should also be noted that there is a growing body of evidence suggesting the effectiveness of alternative therapeutic approaches in the treatment of various disorders, including hypertension. Thus, epidemiological evidence suggests the existence of a negative correlation between consumption of polyphenol-rich foods (fruits, vegetables, cocoa, etc.) or beverages (wine, especially red wine, grape juice, tea, etc.) and the incidence of cardiovascular disease [5, 6].* Thymus serpyllum* L. (wild thyme, TE) has traditionally been used as a spice plant, whose aqueous extract is rich in the polyphenolic compounds [7] that are considered to be responsible for their antioxidant effects. Also, the antihypertensive effect of essential oils from Chinese medicinal plants was confirmed in experimental studies [8]. Similarly, water extracts of plants from Lamiaceae family, rich in phenolic acids, decreased systolic blood pressure after subcutaneous administration in conscious stroke-prone spontaneously hypertensive rats [9] and inhibit rabbit lung angiotensin I-converting enzyme* in vitro* [10]. In addition, our previous study showed that aqueous extract obtained from TE induces powerful NO-independent systemic vasodilatation in spontaneously hypertensive rats (SHR) [7].

On the other hand, it is well known that reactive oxygen species (ROS), amongst other factors, play an important role in the development of hypertension in many experimental models, as well as in patients with essential, renovascular, and malignant hypertension [11]. Several experiments in which the expression of heme oxygenase-1 (HO-1), inducible form of the rate-limiting enzyme in the degradation of heme [12], was upregulated by different modulators or by gene transfer suggested that HO-1 participates in defence mechanisms against agents that induce oxidative injury [13]. Heme oxygenase cleaves the heme ring to form the water-soluble 1-carbon fragment as carbon monoxide (CO), iron, and a biliverdin [14], which is reduced by biliverdin reductase to bilirubin (lipophilic linear tetrapyrrole), a compound with potent antioxidant capacity, abundant in blood plasma [15]. Further studies confirmed the circulating forms of bilirubin, such as free bilirubin, albumin-bound bilirubin, conjugated bilirubin, and unconjugated bilirubin as effective scavengers of peroxyl radicals able to protect human low-density lipoprotein against peroxidation [13]. The CO, generated in equimolar concentrations to biliverdin during heme oxidation by HO, like NO, inhibits platelet aggregation and acts as a vasodilator when bioavailability of NO is limited [16]. This relaxation of vascular smooth muscle cells results from activation of pathways, including the stimulation of soluble guanylyl cyclase, opening of calcium activated K^+^ channel, inhibition of cytochrome P450-dependent monooxygenase, or blocking the production of constrictor substances like endothelin [17]. Furthermore, the increased ROS production that was observed in hypertensive animals and humans could be reduced by treatment with superoxide dismutase (SOD) mimetics or antioxidants, resulting in the improvement of vascular and renal function, regression of vascular remodelling, and reduction of blood pressure [11]. Considering all the above, we hypothesized that TE regulates blood pressure and oxidative stress of SHR through a mechanism that could involve HO-1.

Therefore, the aim of the present study was to evaluate the ability of TE treatment to upregulate the expression and activity of inducible form of HO-1 and its correlation with antihypertensive as well as antioxidant responses of SHR.

## 2. Material and Methods

### 2.1. Thyme Extract Preparation

Dry thyme extract (TE) from* Thymus serpyllum* L. was prepared as described previously [7]. Briefly, TE was extracted by pouring 200 mL of boiled distilled water over the herbal samples (10 g) at room temperature, filtered through a tea strainer, and freeze-dried. The yield of freeze-dried TE amounted to 1.67% (w/w). The total polyphenols content of the corresponding freeze-dried TE exhibited 20.08 mg GAE/100 mg of TE, while the HPLC analysis of the polyphenolic profile of the obtained extract showed rosmarinic (4.30 mg/100 mg of freeze-dried TE) and caffeic acids (0.08 mg/100 mg of freeze-dried TE) as predominant TE phenols. The antioxidant capacity of TE evaluated by the FRAP and ABTS assays amounted to 1.66 mmol Fe(II)/100 mg and 86.1 *μ*mol Trolox/100 mg of freeze-dried TE, respectively. As the dose-dependent antihypertensive evaluations revealed only the dose of 100 mg/kg freeze-dried TE effective in reducing systolic blood pressure [7], the present study was carried out with this TE dose.

### 2.2. Animals

We used six-month-old male spontaneously hypertensive rats (SHR, descendants of breeders originally obtained through Taconic Farms, Germantown, NY, USA) and normotensive* Wistar* (W) rats, bred at the Institute for Medical Research, University of Belgrade, Serbia, weighing about 300 g. They were maintained in temperature and humidity controlled rooms on a twelve-hour light-dark cycle.

The experimental protocol was approved by the Ethic Committee of the Institute for Medical Research, University of Belgrade, Serbia (number 0312-1/10), according to the National Law on Animal Welfare.

Rats were divided in 4 groups: two control groups, normotensive W rats (W-C) and SHR-C, received vehicle (0.2 mL saline), and two treated groups, W-TE and SHR-TE, received i.v. bolus of TE (100 mg/kg b.w. dissolved in 0.2 mL saline).

### 2.3. Systemic Haemodynamic Measurements

For the direct haemodynamic measurements, after bolus injection of TE or vehicle, all rats were anesthetized with 35 mg/kg b.w. sodium pentobarbital, intraperitoneally. Mean arterial pressure (MAP) and heart rate (HR) were measured through a femoral artery catheter (PE–50, Clay-Adams Parsippany, NY, USA), connected to a physiological data acquisition system (Cardiomax III-TCR, Columbus Instruments, Columbus, OH, USA). A jugular vein was cannulated with polyethylene tubing PE-50 for the injection of solutions. The left carotid artery was catheterized with a thermo sensor, which was coupled to Cardiomax III for the determination of cardiac output. The second end of thermocouple sensor was placed in cold saline. Following 20 min for stabilization after surgery, rats were given a bolus injection of TE or vehicle, and haemodynamic parameters were recorded 30 minutes after injection. Cardiac output was normalized to body weight and expressed as cardiac index (CI, mL/min/kg). Total peripheral vascular resistance (TPVR, mmHg × min × kg/mL) was calculated from MAP and CI (assuming that the mean right atrial pressure is zero).

### 2.4. Regional Haemodynamic Measurements

For regional blood flow measurements left carotid artery was gently separated from the surrounding tissue. An ultrasonic flow probe, 1RB (internal diameter = 1 mm) was placed around the artery and total carotid blood flow (CBF) was recorded using a Transonic T106 Small Animal Flowmeter (Transonic T106 Small Animal Flowmeter, Transonic System Inc., Ithaca, New York). After abdominal incision renal artery preparation was utilized and renal blood flow (RBF) was recorded. Vascular resistance in these two vascular beds (CVR and RVR) was calculated by dividing MAP with total blood flow through respective blood vessel, normalized for the body weight, and expressed as mmHg min kg/mL.

### 2.5. Plasma Sample and Tissue Isolation

Blood samples obtained by puncture of the abdominal aorta were collected under anaesthesia, 30 minutes after TE or vehicle application, into tubes containing lithium-heparin (Li-heparin, Sigma, USA) as an anticoagulant. The plasma was separated by centrifugation at 4000 rpm for 20 min. After plasma removing, the remaining erythrocytes were rinsed three times, aliquoted, and stored at −80°C. Liver and kidney tissues were removed immediately on ice, rinsed with cold saline, weighed, and then cut into portions, frozen in liquid nitrogen, and stored at −80°C for the later estimation of protein content and enzymatic antioxidant defence.

### 2.6. Enzyme-Linked Immunosorbent Assay for Heme Oxygenase-1

Plasma and liver HO-1, an inducible heme degrading enzyme with antioxidant properties, was detected and quantified using commercially available, rat specific, enzyme-linked immunosorbent assay kit (HO-1, rat, EIA. ADI-EKS-810A, Enzo Life Sciences International, Inc.). Plasma, previously stored at −20°C, was defrosted and in accordance with the manufacturer's recommendations prepared for analysis. On the day of analysis, tissue was homogenized and prepared for the assay procedure according to the manufacturer's instructions. All samples, including standard, were assayed in duplicate. The intensity of the colour was measured in a microplate reader at 450 nm. HO-1 concentrations from the sample were quantitated by interpolating absorbance readings from a standard curve generated with the calibrated HO-1 protein standard. Values were expressed as ng/mL for plasma HO-1 and as *μ*g/g of liver tissue.

### 2.7. Heme Oxygenase Enzyme Activity

Plasma bilirubin serves as a marker for heme oxygenase activity. Direct bilirubin (BIL-D) and total bilirubin (BIL-T) levels were determined using commercial kits for automatic analyser COBAS INTEGRA 400 plus (Hoffmann-La Roche, Germany).

### 2.8. Superoxide Dismutase, Catalase, and Glutathione Peroxidase Enzyme Activities

Antioxidant enzyme activities of the erythrocytes (e) as well as liver (L) and kidney (k) homogenates were measured by following the spectrophotometric methods: catalase (CAT) was determined as previously described [18], glutathione peroxidase (GPx) was measured according to Paglia and Valentine [19], and superoxide dismutase (SOD) was determined according to McCord and Fridovich [20].

### 2.9. Lipid Peroxidation

For the assessment of lipid peroxidation in plasma (p), liver (L), and kidney (k) lipid peroxide levels were estimated by assaying thiobarbituric acid reactive substances (TBARS) at 540 nm using 2-thiobarbituric acid (4,6-dihydroxy-2-mercaptopyrimidine; TBA, Acros, Organic). An extinction coefficient of 156000 M^−1^ cm^−1^ was used for calculation [21]. The level of p-TBARS was expressed as nmol/mL, while L-TBARS and k-TBARS were expressed as *μ*mol/g tissue.

### 2.10. Statistical Analysis

The data are given as mean ± SEM. Comparisons between the respective groups were made by using* Student's t-tests*. *p* values < 0.05 were considered significant. Correlations between obtained parameters in normotensive as well as in hypertensive rats were also examined, and *p* values < 0.05 were considered significant (Statistica 8.0 for Windows).

## 3. Results

### 3.1. Effects of TE on Haemodynamic Parameters

**Figure 1:**
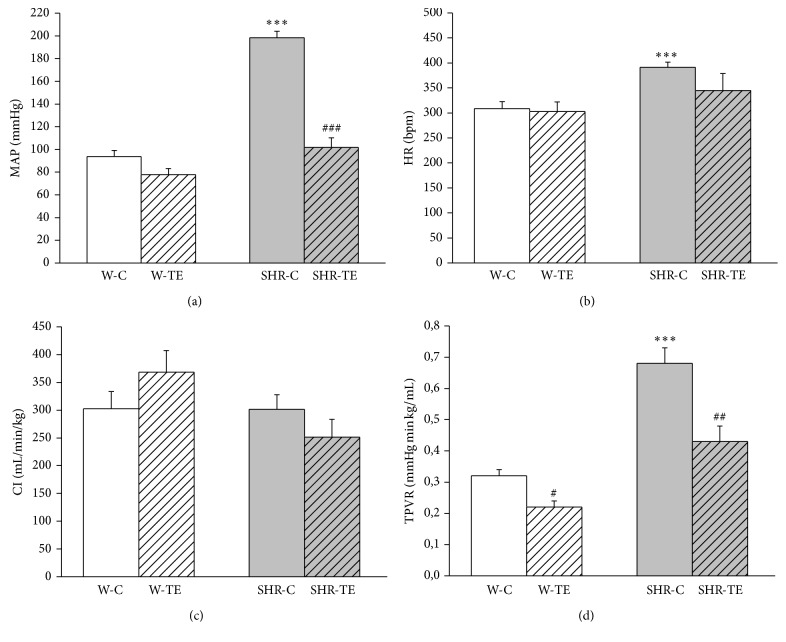
Haemodynamic parameters: (a) mean arterial pressure (MAP), (b) heart rate (HR), (c) cardiac index (CI), and (d) total peripheral vascular resistance (TPVR) in experimental groups. W-C, normotensive* Wistar* rats, and SHR-C, spontaneously hypertensive rats, received vehicle. W-TE and SHR-TE groups received thyme extract. ^*∗∗∗*^ *p* < 0.001, the significant difference between SHR-C and W-C; ^###^ *p* < 0.001, ^##^ *p* < 0.01, and ^#^ *p* < 0.05, the significant difference between TE treated and appropriate control group.

Haemodynamic parameters are shown in Figure 1. As expected, MAP, HR, and TPVR of SHR-C were significantly higher compared to the values of these parameters in W-C group (*p* < 0.001), but without changes of CI. Bolus injection of saline solution containing dry thyme extract resulted in a significant decrease of MAP (*p* < 0.001) only in the group of SHR-TE, while TPVR was reduced in both SHR-TE (*p* < 0.01) and W-TE (*p* < 0.001) rats compared to their respective controls (Figures 1(a) and 1(d)) without changes in CI (Figure 1(c)). Injection of TE did not affect HR of both rat strains (Figure 1(b)).

### 3.2. Effects of TE on Regional Haemodynamic Parameters

**Figure 2:**
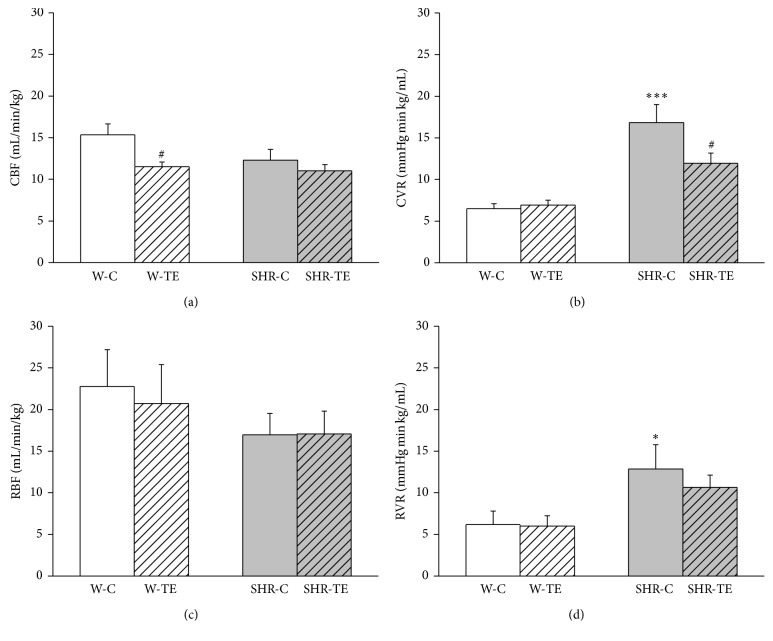
Regional haemodynamic parameters: (a) carotid blood flow (CBF), (b) carotid vascular resistance (CVR), (c) renal blood flow (RBF), and (d) renal vascular resistance (RVR) in experimental groups. W-C, normotensive Wistar rats, and SHR-C, spontaneously hypertensive rats, received vehicle. W-TE and SHR-TE groups received thyme extract. ^*∗*^ *p* < 0.05 and ^*∗∗∗*^ *p* < 0.001, the significant difference between SHR-C and W-C; ^#^ *p* < 0.05, the significant difference between TE treated and appropriate control group.

Regional haemodynamic parameters are presented in Figure 2. The resistance in both vascular beds, carotid and renal artery, were significantly elevated in SHR-C compared to W-C (Figures 2(b) and 2(d), *p* < 0.001 and *p* < 0.05), without changes of respective vascular blood flow (Figures 2(a) and 2(c)). Acute TE treatment led to a decrease of CBF in* Wistar* rats (Figure 2(a), *p* < 0.05), while in the SHR caused a significant reduction of CVR (Figure 2(b), *p* < 0.05). Despite marked reduction of MAP, RVR remained nonsignificantly changed due to acute TE treatment (Figure 2(d)) but still showed a tendency to get closer to W groups.

### 3.3. Effects of TE on Plasma and Liver HO-1 Enzyme Concentration

**Figure 3:**
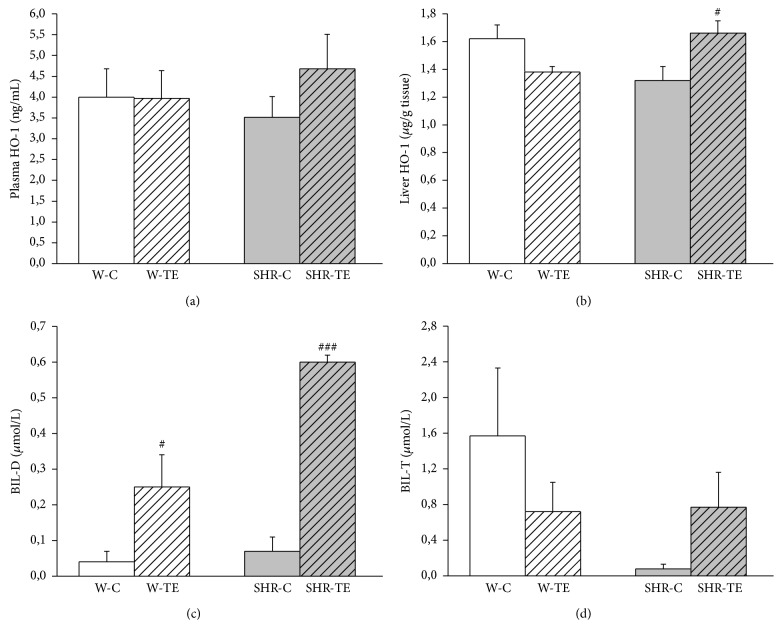
The quantity of (a) plasma and (b) liver heme oxygenase-1 (HO-1), (c) direct bilirubin (BIL-D) in plasma, and (d) total plasma bilirubin (BIL-T) in experimental groups. W-C, normotensive Wistar rats, and SHR-C, spontaneously hypertensive rats, received vehicle. W-TE and SHR-TE groups received thyme extract. ^###^ *p* < 0.001 and ^#^ *p* < 0.05, the significant difference between TE treated and appropriate control group.

We found that plasma HO-1 concentration did not differ significantly between the examined groups, although the value in SHR-TE group was the highest (Figure 3(a)). The expression of liver HO-1 (Figure 3(b)) was lower in hypertensive than in normotensive rats (1.32 ± 0.10 versus 1.62 ± 0.10 mg/g tissue, *p* = 0.0634). TE treatment significantly increased the content of this enzyme in hypertensive rats (SHR-TE, 1.66 ± 0.09, versus SHR-C, 1.32 ± 0.10 mg/g tissue, *p* < 0.05). On the contrary, the application of TE into normotensive rats resulted in a prominent decrease of quantity of this enzyme (W-C: 1.62 ± 0.10 versus W-TE: 1.38 ± 0.04 mg/g tissue, *p* = 0.0507).

### 3.4. Heme Oxygenase Enzyme Activity

Acute TE treatment significantly increased plasma BIL-D level in both rat strains (SHR-TE versus SHR-C: *p* < 0.001; W-TE versus W-C: *p* < 0.05, Figure 3(c)).  Figure 3(d) shows that the plasma BIL-T content was not significantly different in W rats. However, the decreased BIL-T of hypertensive rats compared to age-matched normotensive rats was significantly reversed by TE treatment (SHR-TE versus SHR-C: *p* < 0.05).

### 3.5. Effects of TE on Plasma, Liver, and Kidney Lipid Peroxidation

**Figure 4:**
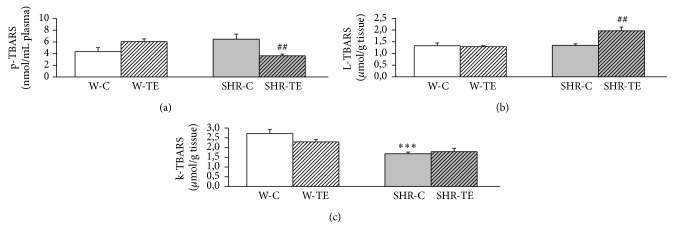
The quantity of (a) plasma (p), (b) liver (L), and (c) kidney (k) thiobarbituric acid reactive substances (TBARS) in experimental groups. W-C, normotensive Wistar rats, and SHR-C, spontaneously hypertensive rats received vehicle. W-TE and SHR-TE groups received thyme extract. ^*∗∗∗*^ *p* < 0.001, the significant difference between SHR-C and W-C; ^##^ *p* < 0.01, the significant difference between TE treated and appropriate control group.

SHR-C had higher p-TBARS then W-C rats (*p* = 0.0772, Figure 4(a)). Acute TE treatment significantly reduced the level of p-TBARS in SHR-TE group compared to SHR-C (*p* < 0.01, Figure 4(a)). On the contrary, in normotensive rats the value of p-TBARS became almost significantly elevated in response to TE treatment (*p* = 0.0625, Figure 4(a)). Liver homogenate TBARS levels did not differ amongst W-C, SHR-C, and W-TE groups. However, L-TBARS was markedly increased in SHR-TE than that in SHR-C group (*p* < 0.01Figure 4(b)). The kidney TBARS level was significantly lower in SHR-C group compared to the W-C (*p* < 0.001Figure 4(c)), and TE had no effects on it, in either hypertensive or normotensive rats.

### 3.6. Effects of TE on Antioxidant Enzymes Activities in Erythrocytes, Liver, and Kidney

**Figure 5:**
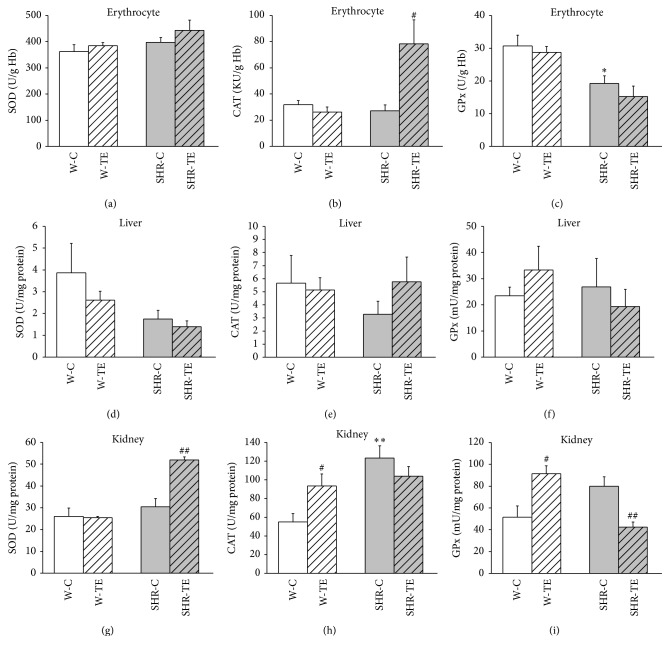
Antioxidant enzyme activities of (a), (b), and (c) erythrocytes, (d), (e), and (f) liver and (g), (h), and (i) kidney in experimental groups. W-C, normotensive* Wistar* rats, and SHR-C, spontaneously hypertensive rats, received vehicle. W-TE and SHR-TE groups received thyme extract, superoxide dismutase (SOD), catalase (CAT), and glutathione peroxidase (GPx). ^*∗*^ *p* < 0.05 and ^*∗∗*^ *p* < 0.01, the significant difference between SHR-C and W-C; ^#^ *p* < 0.05 and ^##^ *p* < 0.01, the significant difference between TE treated and appropriate control group.

SOD, CAT, and GPx enzyme activities in the erythrocytes, liver, and kidney from all experimental animals are shown in Figure 5. There were no differences in erythrocytes, liver, and kidney SOD activities between SHR-C and W-C rats (Figures 5(a), 5(d), and 5(g)). TE treatment significantly increased the kidney SOD activity in the SHR (SHR-TE versus SHR-C: *p* < 0.001), but not in the W rats (Figure 5(g)). Erythrocytes and liver CAT activity (Figures 5(b) and 5(e)) was unchanged in SHR-C as compared to W-C rats, but the treatment has led to the increases of erythrocytes CAT activity only in SHR-TE compared to SHR-C (*p* < 0.05). In contrast, CAT activity in kidney (Figure 5(h)) was found to be increased in both W-TE and SHR-C compared to W-C (*p* < 0.05, *p* < 0.001, resp.). GPx activity from the erythrocytes of SHR-C was significantly lower compared to W-C (*p* < 0.01, Figure 5(c)). Bolus injection of TE induced a significant elevation of kidney GPx activities in normotensive rats (*p* < 0.05, Figure 5(i)). The opposite effect was found in kidney homogenates of SHR (Figure 5(e)). Namely, the activity of GPx was significantly reduced in SHR-TE compared to SHR-C (*p* < 0.01). In the liver homogenates the activity of this antioxidant enzyme was unchanged in both W-TE and SHR-TE groups compared to their controls (Figure 5(f)).

### 3.7. Correlation Analysis of Obtained Parameters

In SHR, vascular resistance of the systemic circulation was found to be positively correlated in relation to MAP (*r* = 0.7546, *p* = 0.005), whereas in normotensive rats the negative correlation of TPVR and CI (*r* = −0.8027, *p* = 0.002) was observed.

Correlation between haemodynamic parameters and oxidative status is shown in Table 1. MAP exhibits a significant positive correlation with p-TBARS. Also, MAP and TPVR showed strong negative correlation with regard to L-TBARS and HO-1 expression and activity in hypertensive but not in normotensive rats. In addition, in the group of hypertensive rats, we found a significant positive intercorrelation between L-TBARS, quantity of plasma and liver HO-1, and bilirubin concentrations, followed by strong and negative intercorrelation between these parameters with p-TBARS, while in the kidney we have not found a correlation between the examined parameters (Table 2).  Table 3 represents the correlation of antioxidant enzyme activity and systemic haemodynamic parameters, with oxidative status in hypertensive and normotensive rats.

## 4. Discussion

Earlier, we suggested that a powerful vasodilator molecule, nitric oxide, is not liable for normalization of blood pressure in TE treated SHR [7].

Here, we hypothesized that induction of HO-1, due to TE treatment, may contribute to powerful blood pressure-lowering effect and reduction of systemic oxidative stress in SHR. In support of our hypothesis are the results by Jin et al. that identified rosmarinic acid as an inducer of HO-1 expression by increasing ROS production* in vitro* [22]. Further evidence identifies derivate of caffeic acid, caffeic acid phenethyl ester, as potent HO-1 inducer that can be used to markedly increase heme oxygenase activity in astrocytes [23]. To our knowledge, the present study is the first showing TE-induction of HO-1* in vivo*, in hypertensive rats. Considering all the above and the composition of used TE, we suggest that this strong induction of HO-1 in SHR represents the response of the liver to TE-induced increase of ROS production. This assumption is supported with significant positive correlation between liver ROS measured by TBARS and the level of HO-1 in hypertensive rats.

In the present study, wild thyme induced significant and pronounced systemic vasorelaxation in both hypertensive and normotensive rats compared to vehicle, but only in hypertensive rats did such relaxation significantly and positively correlate with markedly reduced mean arterial pressure. As expected, results from SHR showed increased TPVR, CVR, and RVR accompanied with significant elevation of blood pressure and HR, without changes of CI, CBF, or RBF compared to* Wistar* rats. Our results in hypertensive rats treated with TE are in favour of previously obtained findings [24–26] that plant polyphenols decrease arterial pressure in SHR. Some studies showed that upregulating the HO/CO system lowers BP in young (8 weeks) but not in adult (20 weeks) spontaneously hypertensive rats (SHR) [27]. Other studies have demonstrated that either acute or chronic administration of HO-1 inducer was able to normalize blood pressure in SHR and that heme administration decreased blood pressure in SHR, while HO inhibitors produced an increase in systemic arterial pressure, even in normotensive rats [28]. The induction of HO-1 that we found in hypertensive rats was in significant negative correlation with the TPVR, indicating that HO-1 generated CO could be accountable for the intensive systemic vasorelaxation and the decrease of blood pressure that we observed in SHR-TE group. These results are in accordance with reports in which, like NO, HO-derived CO serves as a vasodilator to lower blood pressure, regardless of whether it operates via cGMP-dependent or cGMP-independent pathways, thus explaining a number of the potential actions of CO regarding the pathogenesis of cardiovascular diseases [28]. This vasodilator effect may also contribute to the improvement of regional haemodynamics in the carotid artery of hypertensive rats.

The increased iron concentration produced by the HO-1 activity is believed to cause the increased expression of ferritin and ferritin synthesis, which serves to sequester iron, a potent oxidant for cells [28]. Here, we did not determine ferritin content, but there is evidence that tea polyphenols could act as metal chelators [29] and therefore could remove ferrous ions, thereby protecting cells against metal ion-induced oxidative stress.

The next product of heme metabolism, biliverdin, becomes cleaved by biliverdin reductase to bilirubin, a chain-breaking antioxidant that acts as a lipid peroxyl radical (ROO·) scavenger [16]. Bilirubin is toxic in high concentration; however, it prevents adhesion molecule expression and neutrophil adhesion and inhibits ROS and NADPH oxidase activity in biological systems [28]. Serum bilirubin circulates in the bloodstream in forms of direct and indirect bilirubin and has potent endogenous antioxidant properties that are found to have inverse associations with cardiovascular disease [30]. At physiologic oxygen pressure, bilirubin surpasses *α*-tocopherol, the most potent protector against lipid peroxidation. Increased levels of serum TBARS (a by-product of lipid peroxidation) have been previously reported in SHR [31], as well as in patients with hypertension and metabolic syndrome [32], ischemic heart disease [33], peripheral arterial disease [34], and diabetes mellitus [35]. In the present study, along with slight plasma and marked liver HO-1 elevation, plasma level of BIL-D became increased and was followed by reduction of plasma lipid peroxidation in SHR received TE. Also, there was significant negative intercorrelation between the p-TBARS and liver HO-1, as well as BIL-D, confirming the antioxidant defence properties of these endogenous products.

Interestingly, the liver HO system was nearly significantly suppressed in W-TE rats and that might be a possible reason for a moderate increase of plasma lipid peroxidation in this group.

The present study demonstrated almost similar SOD, CAT, and GPx enzyme activity in the liver of all experimental groups, which indicates that these antioxidant enzymes are not crucial for the oxidative status of the liver. Reduced activity of erythrocyte GPx from SHR in comparison to W rats failed to become corrected with TE, but TE induced the enhancement of erythrocyte CAT activity almost threefold and therefore protected SHR against hydrogen peroxide induced systemic oxidative stress. A similar kidney SOD activity that we found in SHR and W rats was in accordance with data obtained by Fortepiani and Reckelhoff [36] who showed that the expression of kidney Mn-SOD and Cu and Zn-SOD was similar in WKY and SHR despite higher ability of hypertensive kidney to produce superoxide. Also, they showed 30% lower expression of GPx and CAT in SHR than WKY and that WKY had increased expression of these enzymes in response to oxidative stress. We did not measure the expression of previously mentioned enzymes, but the activity of k-SOD that we observed was higher after TE in SHR, but not in W rats. At the same time, k-CAT and k-GPx enzyme activity was higher about 2-fold in hypertensive than in normotensive kidney. Further, we found the TE-induced increase of kidney CAT and GPx enzyme activity in normotensive rats. These data indicate that the increased activity of the k-CAT and k-GPx could have been a compensatory mechanism for the prevention of moderate plasma lipid peroxidation that we obtained in W-TE rats.

On the other hand, we found decreased activity of the kidney GPx enzyme in hypertensive rats after TE. Consequently, serum concentrations of bilirubin were high enough to account for a substantial portion of the total antioxidant capacity of serum [15], and bilirubin might alleviate oxidant stress in the blood [14]; we could assume that bilirubin ROO· scavenging activity in our study is sufficient to avoid oxidant stress in the hypertensive kidney. In addition to the direct effects of TE on HO system, the increased k-SOD activity due to the TE injection that we observed in hypertensive rats could be accountable for normalization of BP. Indeed, k-SOD was in significant negative correlation with MAP and TVR in this rat strain, but not in normotensive rats. Further, the increased k-SOD activity was in significant negative association with GPx that in turn negatively correlates against bilirubin, thus emphasizing the bilirubin (regardless of whether it is total or direct) responsible for plasma antioxidant defence.

The numerous polyphenolic substances and other chemical compounds that were found in the plants from the Lamiaceae family were speculated to account for the beneficial effects of these plant extracts on the cardiovascular system. However, the results of this experimental animal study indicate that strong and significant hypotensive and antioxidative activity of aqueous extract from* Thymus serpyllum* L. in hypertensive rats, at least partially, resulted due to targeting heme oxygenase system. Besides rosmarinic acid, used plant extract contains caffeic acid and quercetin and luteolin in trace [7]; thus further studies are needed to elucidate which of these polyphenolic compounds is responsible for those beneficial effects of TE or whether they are a consequence of their synergistic action.

## Figures and Tables

**Table 1:** The correlation between haemodynamic parameters and oxidative status in hypertensive (*n* = 12) and normotensive rats (*n* = 12).

	L-TBARS		p-TBARS		k-TBARS		L-HO-1		p-HO-1		BIL-D		BIL-T	
	W	SHR	W	SHR	W	SHR	W	SHR	W	SHR	W	SHR	W	SHR
MAP	0.2830 *p* = 0.373	−0.7060 *p* = **0.010**	−0.0489 *p* = 0.880	0.6251 *p* = **0.030**	0.2371 *p* = 0.458	−0.1796 *p* = 0.577	0.4435 *p* = 0.149	−0.6159 *p* = **0.033**	−0.4965 *p* = 0.101	−0.3846 *p* = 0.217	−0.3628 *p* = 0.246	−0.9249 *p* = **0.000**	0.4006 *p* = 0.197	−0.6398 *p* = **0.025**
TPVR	−0.1109 *p* = 0.732	−0.7578 *p* = **0.004**	−0.1013 *p* = 0.754	0.4923 *p* = 0.104	0.3308 *p* = 0.294	−0.0962 *p* = 0.766	0.3023 *p* = 0.340	−0.4951 *p* = 0.102	0.1118 *p* = 0.730	−0.6518 *p* = **0.022**	−0.5295 *p* = 0.077	−0.7001 *p* = **0.011**	0.2704 *p* = 0.395	−0.4377 *p* = 0.155

SHR: spontaneously hypertensive rats that received vehicle or thyme extract; W: *Wistar* rats that received vehicle or thyme extract; MAP: mean arterial pressure; TPVR: total peripheral vascular resistance; L-TBARS, p-TBARS, and k-TBARS: liver thiobarbituric acid reactive substance, plasma thiobarbituric acid reactive substance, and kidney thiobarbituric acid reactive substances; quantity of L-HO-1 and p-HO-1: liver and plasma heme oxigenase-1 enzyme; BIL-D: direct bilirubin; BIL-T: total bilirubin. Marked correlations are significant at *p* < 0.050.

**Table 2:** The correlation of oxidative stress parameters with heme oxigenase-1 expression and activity in hypertensive (*n* = 12) and normotensive rats (*n* = 12).

	L-HO-1		HO-1		BIL-D		BIL-T	
	W	SHR	W	SHR	W	SHR	W	SHR
L-TBARS	0.0775 *p* = 0.811	0.6284 *p* = **0.029**	−0.1981 *p* = 0.537	0.6444 *p* = **0.024**	0.0034 *p* = 0.992	0.7475 *p* = **0.005**	0.2548 *p* = 0.424	0.5980 *p* = **0.040**
p-TBARS	−0.2623 *p* = 0.410	−0.7715 *p* = **0.003**	−0.0377 *p* = 0.908	−0.3397 *p* = 0.280	0.0890 *p* = 0.783	−0.7623 *p* = **0.004**	0.0614 *p* = 0.850	−0.5674 *p* = 0.054
k-TBARS	0.6801 *p* = **0.015**	0.4457 *p* = 0.147	0.1703 *p* = 0.597	0.4521 *p* = 0.140	−0.4280 *p* = 0.165	0.0775 *p* = 0.811	0.2146 *p* = 0.503	0.4398 *p* = 0.153

SHR: spontaneously hypertensive rats that received vehicle or thyme extract; W: *Wistar* rats that received vehicle or thyme extract; L-TBARS, p-TBARS, and k-TBARS: liver thiobarbituric acid reactive substance, plasma thiobarbituric acid reactive substance, and kidney thiobarbituric acid reactive substances; quantity of L-HO-1 and p-HO-1: liver and plasma heme oxigenase-1 enzyme; BIL-D: direct bilirubin; BIL-T: total bilirubin. Marked correlations are significant at *p* < 0.050.

**Table 3:** The correlation of antioxidant enzymes activity and systemic haemodynamic parameters, with oxidative status in hypertensive (*n* = 12) and normotensive rats (*n* = 12).

	e-CAT		k-SOD		k-CAT		k-GPx	
	W	SHR	W	SHR	W	SHR	W	SHR
MAP	0.3742 *p* = 0.231	−0.6178 *p* = **0.032**	−0.3627 *p* = 0.247	−0.7413 *p* = **0.006**	−0.6051 *p* = **0.037**	0.2260 *p* = 0.480	−0.3523 *p* = 0.261	0.7480 *p* = **0.005**
TPVR	0.3559 *p* = 0.256	−0.6041 *p* = **0.038**	0.2728 *p* = 0.391	−0.7791 *p* = **0.003**	−0.2638 *p* = 0.407	0.0124 *p* = 0.969	−0.3770 *p* = 0.227	0.5628 *p* = 0.057
L-TBARS	−0.0027 *p* = 0.993	0.6543 *p* = **0.021**	−0.1804 *p* = 0.575	0.7807 *p* = **0.003**	0.0896 *p* = 0.782	−0.1624 *p* = 0.614	0.0496 *p* = 0.878	−0.4627 *p* = 0.130
p-TBARS	0.0818 *p* = 0.800	−0.3587 *p* = 0.252	−0.5557 *p* = 0.061	−0.6479 *p* = **0.023**	0.3237 *p* = 0.305	0.2503 *p* = 0.433	0.2615 *p* = 0.412	0.2261 *p* = 0.480
k-TBARS	0.6637 *p* = **0.019**	−0.2964 *p* = 0.350	−0.0921 *p* = 0.776	0.3710 *p* = 0.235	−0.2610 *p* = 0.413	−0.0555 *p* = 0.864	−0.3946 *p* = 0.204	−0.2397 *p* = 0.453
BIL-D	−0.4345 *p* = 0.158	0.6818 *p* = **0.015**	−0.2737 *p* = 0.389	0.7989 *p* = **0.002**	0.1852 *p* = 0.564	−0.2638 *p* = 0.407	0.3991 *p* = 0.199	−0.6828 *p* = **0.014**

SHR: spontaneously hypertensive rats that received vehicle or thyme extract; W: *Wistar* rats that received vehicle or thyme extract; MAP: mean arterial pressure; TPVR: total peripheral vascular resistance; L-TBARS, p-TBARS, and k-TBARS: liver thiobarbituric acid reactive substance, plasma thiobarbituric acid reactive substance, and kidney thiobarbituric acid reactive substance; BIL-D: direct bilirubin; e-CAT and k-CAT: erythrocyte catalase and kidney catalase; k-SOD: kidney superoxide dismutase; k-GPx: kidney glutathione peroxidase. Marked correlations are significant at *p* < 0.050.
